# Author Correction: Nonequilibrium Magnetic Oscillation with Cylindrical Vector Beams

**DOI:** 10.1038/s41598-020-71839-5

**Published:** 2020-09-01

**Authors:** Hiroyuki Fujita, Masahiro Sato

**Affiliations:** 1 Institute for Solid State Physics, University of Tokyo, Kashiwa, 277-8581 Japan; 2 Department of Physics, Ibaraki University, Mito, Ibaraki 310-8512 Japan

Correction to: *Scientific Reports* 10.1038/s41598-018-33651-0, published online 24 October 2018

When the system is paramagnetic or has a collinear magnetic order, the U(1) rotation symmetry can prohibit for conduction electrons to change the spin polarization without affecting the rest of the system. In this case, therefore, we may need a strong spin-orbit coupling or electron-phonon coupling through which the angular momentum of conduction-electron spins decays and transfers to the environment. If a strong Hund’s coupling exists in a U(1)-symmetric case, the total magnetization of conduction and localized electrons remains conserved while that of conduction electrons can relax and transfer to the localized moment. The field-induced change in the conduction-electron spin is rather small so that the angular momentum is transferred to the localized moments, but the spin texture would be almost unchanged within the timescale of the incident pulse magnetic field. In such a case, Kerr or Faraday effects, which measure the total magnetization, are not useful as the total magnetization is unchanged, but an ultrafast measurement of optical conductivity could observe the CVB-driven oscillation of the conduction electrons.

